# Factors associated with alcohol reduction in harmful and hazardous drinkers following alcohol brief intervention in Scotland: a qualitative enquiry

**DOI:** 10.1186/s12913-017-2093-7

**Published:** 2017-03-08

**Authors:** Jean M. McQueen, Claire Ballinger, Tracey E. Howe

**Affiliations:** 1 Institute of Health and Wellbeing, MRC/CSO Social and Public Health Sciences Unit, University of Glasgow, Glasgow, UK; 2 Faculty of Health Sciences, University of Southampton, Southampton, UK; 3 School of Health & Life Sciences, Glasgow Caledonian University, Glasgow, UK

**Keywords:** Alcohol, Brief intervention, Realist synthesis, Behaviour change, Motivational counselling, General Hospital, Mixed methods

## Abstract

**Background:**

Alcohol Brief Intervention (ABI) uses a motivational counselling approach to support individuals to reduce excessive alcohol consumption. There is growing evidence on ABI’s use within various health care settings, although how they work and which components enhance success is largely unknown. This paper reports on the qualitative part of a mixed methods study. It explores enablers and barriers associated with alcohol reduction following an ABI. It focuses on alcohol’s place within participants’ lives and their personal perspectives on reducing consumption. There are a number of randomised controlled trials in this field though few ABI studies have addressed the experiences of hazardous/harmful drinkers. This study examines factors associated with alcohol reduction in harmful/hazardous drinkers following ABI.

**Methods:**

This qualitative study was underpinned by a realist evaluation approach and involved semi-structured interviews with ten harmful or hazardous alcohol drinkers. Participants (*n* = 10) were from the intervention arm of a randomised controlled trial (*n* = 124). All had received ABI, a 20 min motivational counselling interview, six months previously, and had reduced their alcohol consumption. Interviews were recorded, transcribed verbatim and thematically analysed.

**Results:**

Participants described their views on alcohol, its’ place in their lives, their personal perspectives on reducing their consumption and future aspirations.

**Conclusions:**

The findings provide an insight into participants’ views on alcohol, ABI, and the barriers and enablers to change. Participants described a cost benefit analysis, with some conscious consideration of the advantages and disadvantages of reducing intake or abstaining from alcohol. Findings suggest that, whilst hospital admission can act as a catalyst, encouraging individuals to reflect on their alcohol consumption through ABI may consolidate this, turning this reflective moment into action. Sustainability may be enhanced by the presence of a ‘significant other’ who encourages and experiences benefit. In addition having a purpose or structure with activities linked to employment and/or social and leisure pursuits offers the potential to enhance and sustain reduced alcohol consumption.

**Trial registration:**

Trial registration number TRN NCT00982306 September 22nd 2009.

## Background

Alcohol is a major public health concern. Across Scotland, alcohol related deaths in 2014 were 1.4 times higher than they were in the 1980s [1]. World-wide alcohol consumption is a causal factor in more than 200 disease and injury conditions including mental and behavioural disorders, liver cirrhosis and some cancers [2]. As alcohol consumption levels and associated harms have increased, so too has the interest in alcohol brief intervention [3]. Alcohol Brief Intervention (ABI) consists of a motivational counselling approach involving a time limited, one to one intervention. Its focus is on changing behaviour and enhancing an individual’s ability to reduce consumption. Evidence from two systematic reviews suggests that ABI can support individuals in reducing their alcohol consumption [4, 5]. Within emergency and general hospital care, staff frequently encounter individuals who misuse alcohol and ABI is often delivered within these settings [3]. Whilst there is growing evidence on the impact of ABIs, the views of recipients on how they work and which components are likely to enhance success are largely unknown. An evaluation of the mechanisms of change in motivational interviewing, the first review in this area, assessed 19 studies and highlighted the need for more research to examine questions on how this treatment actually works [6]. In addition, a systematic review also focused on the mechanisms of change in motivational interviewing concluded whilst most studies to date were quantitative, future qualitative studies could deepen our understanding [7]. Publications on ABI in primary care similarly highlight that, despite the numerous ABI research trials, there are still difficulties in identifying the active ingredients of successful brief interventions [8].

To date, few ABI studies have focused on the participants’ experiences as they grapple with the issues involved in reducing or abstaining from alcohol. A search of the literature revealed three potentially relevant studies and one unpublished doctoral thesis [6, 9–11]. Only one study conducted qualitative interviews of participants’ experiences and this study did not focus on ABI per se, but explored 12 professional men’s experiences of alcohol [11]. The three remaining studies, a literature review evaluating the mechanism of change in motivational interviewing [6], a study investigating the impact of change plans in motivational interviewing [9] and one focusing on the interpersonal skill of the therapist [10] did not provide information on the participants’ experiences. That said, within these studies there is consensus on the need to advance knowledge on the process and mechanism of change, in particular, the ‘how’, ‘why’ and ' for whom’ ABIs work [6, 12]. The objective of this study was to investigate participants’ views on alcohol, ABI, and the barriers and enablers to change. It provides an insight into the experience of hazardous and harmful drinkers.

## Methods

This study used a qualitative approach to explore participants’ perspectives on the process of change following ABI. Individuals reducing their alcohol consumption are likely to contend with a complex interplay between, for example, culture, tradition and life events, which, in addition to the ABI, may influence drinking behaviours.

The qualitative approach used for this study was underpinned by a realist evaluation philosophical stance. This approach emphasizes both theoretical thinking and empirical evidence to explain how the intervention being studied works for whom and in what circumstances [13]. Realism uses the mechanism, context and outcome pattern to explain and understand how health interventions work [13].

### Sample

The participants were all hazardous/harmful drinkers who had been admitted to the orthopaedic wards of a General Hospital in Glasgow, where the ABI was delivered. Participants were admitted for a variety of reasons including chest pain, fractures, pancreatitis and infections. Those with both alcohol related and non-alcohol related diagnosis were included. All participants were in the intervention arm of a randomised controlled trial comparing ABI with usual hospital care (*n* = 124, 63 in intervention group and 61 in control group reported in detail elsewhere) [3]. The sample comprised one of convenience, whereby blinding was broken for 12 participants from the intervention group. This sampling technique meant minimal interference with blinding of the assessor for the RCT, as the same researcher completed both qualitative and quantitative follow up in the mixed method study. Of the 12 participants, ten reported alcohol consumption reductions as measured using the Fast Alcohol Screening Tool (FAST) [14] and/or grams of alcohol consumed per week. These respondents were invited to take part in the semi-structured interviews. Participants indicated through their retrospective drinking diary that they had reduced or ceased alcohol consumption in the 6 months following hospital discharge.

### Data collection procedure

**Fig. 1:**
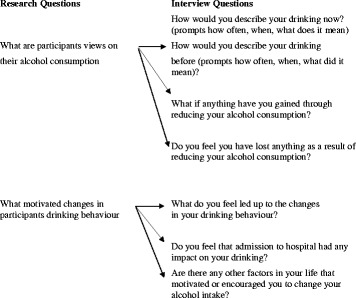
Semi-structured interview schedule. Semi-structured interviews were used to elicit the research participants' experiences of reducing their alcohol consumption, encouraging them to elaborate on their feelings and personal motivation for change

Data were collected during semi-structured telephone interviews. Three key questions guided the interview sessions with additional open ended questions used as prompts (see Fig. 1). This provided the opportunity to explore participants’ experience in greater depth, providing a vivid and illuminating insight into participants’ views on ABI, alcohol consumption and barriers and enablers to change. The interviews involved open questions to deepen the researchers’ understanding of the participants’ experiences of hazardous/harmful drinking and the meaning they attributed to this. They were recorded and transcribed verbatim and lasted on average 25 min.

### Data analysis

The six phases for thematic analysis identified by Braun and Clark [15] were followed; data familiarisation, generation of initial coding, emerging themes, analysis and connecting themes, development and naming of each theme and telling the story of the data in a coherent way.

### Rigour

Continual immersion in the data at all stages before and during the analysis enhanced rigour [15]. Immersion in the data, through reading and re-reading the transcripts, allowed comprehension of its meaning both prior to and during the identification of emergent themes. The credibility of the super-ordinate themes and sub-themes in relation to the data set was ensured through double coding, continual revisions and checking of coded data extracts and transcriptions, individually and collectively, to reflect the meanings evident in the data. Emerging findings were shared and discussed within the research team, to enable assumptions to be challenged and to promote reflexivity.

### Ethics approval

Ethical principles were followed as outlined in the Medical Research Council Good Research Practice Principles and Guidelines [16]. Ethical clearance was obtained from the South Glasgow and Clyde Research Ethics Committee (08/S0710/49). As part of this approval, each participant received a written participant information sheet, advising that participation was voluntary and assuring the person that they could decline to answer any question that they felt uncomfortable with and they were at liberty to withdraw at any time without consequence. The anonymity of the participants was protected by using pseudonyms.

## Results

**Fig. 2:**
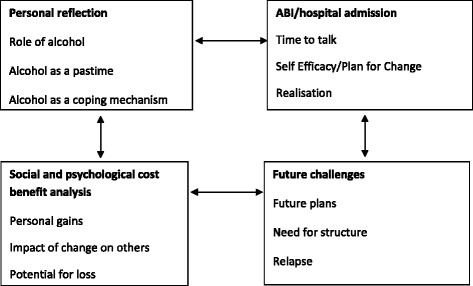
Superordinate Themes. The results show a range of interrelated themes depicting the participants’ experiences of ABI, and the barriers and enablers to behaviour change which in turn influenced decisions to reduce their alcohol consumption

In total nine (9) men and one woman (1) formed the study sample and ranged in age from 23 to 70 years. Analysis revealed a number of enablers and barriers associated with alcohol reduction following ABI. A range of issues emerged iteratively through the analysis, resulting in four super-ordinate themes and a number of sub-themes which explain the participants’ views of alcohol, its place in their lives and personal perspectives on change. The four super-ordinate themes are illustrated with excerpts from the transcripts to illuminate the experience of behavioural change and the meaning of alcohol. They provide details on a range of issues identified, focusing on 1) personal reflection, 2) ABI/hospital admission, 3) cost benefit analysis, 4) future challenges (Fig. 2). Five of the more dominant sub-themes (time to talk, personal gains, impact of change on others, potential for loss and relapse) are explored in more detail to illustrate the depth and range of perspectives.

### Personal reflection

In discussing their experiences, participants had contrasting ways of describing alcohol, reflecting both positive and negative aspects. Some participants described their previous alcohol consumption positively as a pastime, ‘normal behaviour’ and something that could be enjoyed, as a key component of their social lives. Alcohol was viewed as a diversionary form of entertainment, an opportunity for social interaction, or a way to counteract boredom. Colin spoke of previous alcohol consumption as an enjoyable way to relax, describing the atmosphere of the pub and detailing what he liked about it.

*‘Yeah that’s what it was, it’s all about a way to relax and socialise, its good you know the ‘craic’* (fun or enjoyment) *down the pub, it gets me out to meet a few folks you know, have a laugh, put the world to rights* (laughing) *it helped me relax’*

For other participants, alcohol was portrayed as a way to deal with difficult and harrowing life events, using alcohol almost in a self-medicating way, as in the excerpt below where Sharon linked alcohol as a way to numb emotional pain following the death of her mother. Here, the meaning and context of alcohol was different from a weekend pastime and was seen as a much needed coping strategy. Sharon debated her dependence on alcohol, emphasising her strongly held belief that she needed alcohol just to get through the day.

*‘The alcohol helped me face the world. I needed it* (clearing her throat) *well I should say an’ at the time I thought I needed it,* (pause) *well I did need it* (pause) *anyway. I couldn’t face things you know it was a hideous time my mum’s death, but any respite or benefit from alcohol, it was short-lived though, you could say it didn’t help me the next day* (sigh) *I’d feel hungover and even more depressed’*

Participants shared their previous views on alcohol consumption during times when they were drinking heavily. Some, like Sharon, held a firm belief that they needed alcohol to deal with emotional and psychological pain. The death of a close family member featured strongly when participants were recalling when their drinking began. Martin spoke of drinking being a ‘weakness’ and a reaction to the death of his brother, appearing almost angry at himself that he needed alcohol in this way, coupled with a frustration that those around him didn’t understand his use of alcohol to cope. As he described:

*‘Well the drinking* (pause) *it was a weakness and I didn’t know when to stop…… it was mental, drinking from Friday right through to Sunday, I didn’t like them* (family) *getting on at me, we all felt bad about my brother’s death, but I was the one who had to go on the drink, and they just didn’t understand or want to understand that was my way of coping.’*

Here Martin very clearly articulates the meaning and context of alcohol within his life following bereavement, illustrating through personal reflection the complex relationship between alcohol and the death of his brother. As with Sharon, Martin also highlights his alcohol consumption as a coping mechanism. He is also judgemental about this (i.e. describing it as a ‘weakness’), reinforcing that the identification of alcohol as a coping mechanism does not necessarily imply approval. It is possible that the ABI contributed to Martin’s personal reflection on the role alcohol played in his life, although it is likely that a number of factors, such as the accident and subsequent hospital admission, influenced his decision to reduce his alcohol consumption.

Participants convincingly spoke of how they had needed alcohol to help them deal with emotional and psychological pain during difficult times. Admission to hospital was viewed as an opportunity to take stock and reflect on their alcohol consumption.

### ABI and hospital admission

In discussing their experiences, participants spoke about the ABI and hospital admission, and from their narratives it was difficult to make a clear distinction between the two. Therefore, they have both been considered within this one superordinate theme. It is possible that the hospital admission afforded participants time to reflect and the ABI provided the opportunity and permission to explore and reflect on their alcohol use. All of the participants were able to recall their recent admission to hospital and the discussions they had with the clinical researcher about their alcohol consumption (the ABI). Many appeared to value this opportunity and the time to consider what alcohol meant to them.

#### Time to talk (sub-theme)

The participants reported feeling listened to and valued, which appeared beneficial in initiating the change process. Having a personalised plan for change (as was offered in the ABI process) was important and appeared to give, for example, Sam the chance to rationalise how any proposed changes might work for him. Sam acknowledged the ABI delivered in hospital:

*‘Well it was needed* (ABI)*. I needed to talk about what I was doing to myself with the drinking, it gave me the chance to really think about it. It’s maybe something I’d been hiding from all along* (cough) *but the fact that someone else had noticed and took the time to speak to me about it* (pause) *well it just made me think and well* (pause) *I’m glad, I’m really glad because there’s always the chance that I might not have bothered.’*

Sam’s use of the phrase ‘hiding’ appears to indicate that, whilst he was aware that his drinking was excessive, he was to some extent in denial, hiding it from himself. In contrast, Chris, at the time of his hospital admission, did not believe his alcohol consumption was an issue. Chris described his admission to hospital being more of a ‘*wake-up call’*. He spoke of identifying something he had not been aware of previously. It is interesting to note that although Chris appeared to lack awareness, he did not mention any issues or concern at being approached and asked about his alcohol consumption, or in participating in the ABI.

*‘Aye,* (yes) *I suppose being in hospital gave me a bit of a wake-up call you know,* (pause) *hadn’t viewed my drinking as a problem, it’s what I’d always done, but you know had a bit of time to think about things, can be a long day in there* (hospital) *not much happenin’ outside of visiting hours, an’ I was thinking about the panic attacks I’d been having and trying to deal with them better, the fact my drinking was commented on…well it made me think.’*

In many instances being asked about their drinking patterns appeared to be an important first step on the journey to reduce their drinking. Sam and Chris mentioned the importance of acknowledging their current issues with alcohol and, through the ABI, devising a plan of action. ABI appeared to give permission for participants to review their alcohol consumption and explore the possibilities of change. Hospital admission provided the time and space for the personal reflection outlined earlier, with the potential for health-related concerns to escalate the need for change.

### Social and Psychological cost-benefit analysis

Participants focused on the impact they believed alcohol was having on significant areas of their life, akin to a cost benefit analysis, considering factors such as their health, finances, social life and relationships. The narratives suggest participants considered personal gains, the impact of change on others, and weighing this up with the potential for loss.

When considering change, participants emphasised and reviewed these factors, fulfilling the human need to reflect, understand and rationalise before deciding on a course of action, as illustrated in the excerpt below. Steve spoke of ‘weighing up what was important’ whilst in hospital:

*‘Yeah, it* (hospital admission) *made me weigh up what was important* (pause) *not long after I came out of hospital I went to a friend’s 21* ^*st*^ *party and didn’t drink* (pause) *that would never have happened before.’*

When asked what would normally have happened before, Steve reflected:

*‘I’d get really drunk and out of it, start arguing with people around me about daft stuff and just making a fool of myself generally.’*

Consideration of the pros and cons for change appeared to form an important pre-requisite and an instrumental part of these individuals’ quest to reduce or abstain from hazardous and harmful patterns of drinking. It is possible that participants were beginning to imagine what life could be like if they reduced or abstained from alcohol and this may be related to the motivational nature of the ABI or the time afforded to participants whilst in hospital.

#### Personal gains (sub-theme)

Participant narratives focused on the personal gains associated with drinking less alcohol. The perceived consequences of excessive alcohol consumption appeared to play a part, for some, in decision making and identifying the right time for change. Colin compared himself to others he saw returning from the pub and the negative consequences of excessive alcohol consumption he’d observed, believing that these could affect him too. This enhanced his motivation, providing a valid reason to consider change and escalating the priority attached to the changes. As Colin notes:

*‘Well, I was looking out of the window and seeing them all walking down the road buckled* (drunk) *after I came home from hospital an I’m still sort of upright, but I was like I’m no’ that far away from that….and then the rattling* (withdrawal symptoms)*. I never rattled but I couldny have been far away from it, you know what I mean.’*

In recalling personal experience, Colin emphasised he did not want to be like those returning drunk from the pub and this appeared to enhance his motivation for change. Colin’s motivation was fuelled by fear of becoming like those he describes in the excerpt above. In a similar vein, Steve cites health as a factor reinforcing the changes he had made:

*‘Well it’s my health, really it’s much better, I’m diabetic and well my insulin’s now under control* (pause) *I was missing my insulin ' cause I was out of it with the drink my health was all messed up* (pause). *For me when I start drinking I can’t stop at one or two, and then I ended up drinking so much forgetting to do it* (self-injecting insulin) *and then as you know ended up in hospital.’*

Within this sub-theme, participants appear to be outlining how their attitude to alcohol shifted, considering either perceived or real harms, and representing a personalised process weighing up the benefits and drawbacks of reducing or abstaining from alcohol. Factors influencing this appeared to be linked to either actual or perceived negative consequences associated with their alcohol consumption; for example, financial implications or health concerns. This theme is built on further through participants’ reflection on the impact changes in their alcohol consumption had on those around them.

#### Impact of change on others (sub-theme)

The presence of a significant other, someone who cared enough to take an interest in the participant’s alcohol use appeared to be important, and linked to positive change. These factors centred on positive encouragement received through interacting with others. Gaining respect or positive affirmation that changes were beneficial appeared powerful. This created a sense of achievement for participants, since their efforts to reduce alcohol consumption had been recognised and credited by those important in their lives. For Steve, this comprised his family and looking forward to the future they could have together:

*‘For me it wasn’t difficult* (pause) *not really* (pause)*. I was thinking of my family, definitely my family is what made the difference and their future. All I was doing was arguing with the ones I loved and making a right fool of myself. I needed to think about my future and the wee man’s* (sons) *future, things are much better now without the drink….much better, my health’s better and I’m no longer getting into as many arguments.’*

Sam viewed comments from those around him as encouraging behavioural change, as he expressed a sense of pride and support that people had commented on his willpower. As in the case of Steve, Sam’s family saw benefits, with his wife identifying positive advantages to his reduced level of drinking; he was now spending more time with her, enjoying meals out and holidays. This positive impact on a significant other appeared important in sustaining the changes made, as Sam illustrates*:*

*‘Yes for my wife, well, she says she’s got the man back she married. It’s been really good and not just for me as well, for my wife and my daughter, it’s not just me that’s got the benefit….I don’t think anyone thought I could do it* (give up the drink) *though* (laughing)*. Even my daughter has been totally surprised by my willpower.’*

These positive benefits identified by others appeared to function as motivating factors that are not only likely to positively influence change, but also support the sustainability of these changes. However, it is important to note that not all participants viewed their reduced alcohol consumption positively as described in the next section.

#### Potential for loss (sub-theme)

As with many change processes, there was also a sense of loss involved. The balance between perceived losses versus perceived gains appeared not only to influence the decision to change, but also the sustainability of these changes. Participants did not always identify reduced alcohol consumption as a positive change. Both Willie and John, interestingly the only two participants who lived alone, missed the social contact offered by their previous drinking patterns. As Willie described:

*‘Well it’s the social side I miss that’s all, well it’s to pass the time more or less you know, I always like to have a blether* (chat) *and that sort of thing, it’s a good way to meet people.’*

Both participants who reported missing something following their abstinence or reduced alcohol consumption reported that it was not so much the alcohol per se, but what came with it; namely, the opportunity to meet people, have a laugh and almost escape from the pressures in their lives for a short time. John missed the company of his friends and the opportunity to meet and relax over a few pints, highlighting that they (his friends) were not supportive of the changes he had made. This could have an impact on the sustainability of the change, as John reflects:

*‘Ehh well, I miss the company, the chat you know the social side of the pub eh the company of my pals, an’ sometimes* (pause) *you know they’re no’ happy that I’m not drinking* (pause) *but, well I’m not doing it as much as I used to ….it just got into a habit, but I miss it, the company, the chat, but like I said it’s a habit, but you know well* (sigh)*. I just canny relax as well without it.’*

John spoke fondly of his experiences at the pub with his friends and this was what he missed most about drinking. In John’s case, the sustainability of the changes may be jeopardised if he is unable to find something to fill this gap, with some other form of relaxation or social contact.

Tom’s solution was to continue going to the pub or meeting with the friends he used to drink with in order to access the company and social life. He spoke of changing his drinking behaviour by switching to non-alcoholic drinks. As Tom expressed:

*‘For sure, I can go to a normal run of the mill pub, don’t get me wrong, I can still go for the company and not really drink too much at all and have a few non-alcoholic drinks.’*

Tom’s unique solution meant he still gained from the social life associated with the pub. In contrast, Sam had a different experience, reporting his delight at the surprise from his drinking friends when he told them he was not drinking anymore. In contrast to both Willie and John, he appeared to view this change with a positive attitude, a sense of pride and saw his behaviour as something he believed his drinking friends admired. This positive reinforcement may have acted to cement the changes he had made.

*‘I still meet some of the guys from the bowling when I’m out and about and they say “We havn’y seen you for ages, you’ve no been down the club”… I tell them “No, I’m not drinking” and it’s a total surprise, you just see this look and you can tell they’re shocked, and do you know they say “Well done, I wish I could do that”….yeah I think I’ve really surprised a lot of people.’*

However, it is important to note that Sam did not live alone and had the support of his wife unlike John and Willie. This may have influenced Sam’s view of abstaining from alcohol, which clearly differed from John and Willie.

In summarising this super-ordinate theme, then, the process and mechanism involved in behavioural change followed participants’ contemplation of the role alcohol played in their lives, with many using a cost-benefit analysis to highlight what they had gained and what they had lost by reducing or abstaining from alcohol. The process of change appeared to be initiated by focused time for reflection, which may have been prompted by the ABI and enhanced by hospital admission, thus giving participants time to take stock, away from factors that may influence their drinking, within an environment (hospital) where alcohol was not easily available.

### Future challenges

Participants focused on their views of the future, their hopes but also their fears and potential for relapse. As with any behavioural change there is always potential for relapse and participants spoke of protective factors they hoped would guard against this. Tom held a strong belief that having concrete plans for the future focusing on a life with less alcohol would make a difference, as the following narrative illustrates.

*‘If you’ve got something to get yourself sorted out for, well that keeps you going, something to think to the future for, something positive, buying a new car is a goal of mine so I’ve got the added incentive of trying to save money’*

Both Sharon and Colin were unemployed and interestingly both opined it was important to get a job, believing that this would provide more of a purpose and structure to their lives. Both believed that having a job would be a protective factor in their lives as Colin highlights:

*‘Well I’m dying for a job, I’ve had a couple of cuts to my face* (knife wounds)*, ermm so that’s gonna make it hard to get. At the job centre they said”Just lie and say you had surgery you went through a winch crane”…but my whole life’s been based on lies, but you know I’d rather just tell the truth. Even if they were willing to give me a two week trial* (pause)*. I’m no’ even wanting paid, I’ll show them what I can do. Eight hours, you know, spending eight hours in a place* (laughing) *and then eight hours’til you go to bed, you can’t ask more than that.’*

Sharon also alludes to the positive impact which she believes a job could offer her in the excerpt below:

*‘So now I only drink socially* (pause)*, so it’s not that often really. This week all I had was some rosé wine at a barbeque and before that it was April for a friend’s birthday. I had a couple of pints of cider and I was feeling tipsy* (laugh) *and so I just got a taxi home, so for me that’s a big change. I just need to focus on getting a job now…but I’m taking each day as it comes, but I know I need something to get me out of the house and keeping me busy. I’m not really too fussed about what type of work, I’ll take anything I can get. I just need something to keep me busy, a reason to get up every day and get out.’*

For Tom, Sharon and Colin, having a positive goal and something that could take the place of alcohol was important in helping them to sustain changes they had made and maintain their motivation. Similarly, Martin commented on the importance of plans for the future, saying he had not mastered this yet, but recognised he needed plans in place in case of difficult, emotionally charged times ahead. Martin reflects;

*‘I’ve had some really difficult things to deal with…and so yeah it did help* (alcohol*) in some ways, but not in others, I suppose you could say. But I hope I’m through that stage in my life, need to look forward. I don’t have any major plans for the future yet, but I am working on them.’*

The fact that these three participants identified the need for structure appears positive and potentially linked to ABI, and the impact of the personal change plans. However, it is somewhat concerning they still do not have this structure in their lives 6 months down the line.

#### Relapse (sub-theme)

Another sub-theme specifically illustrated by Colin, who had previously tackled a drug addiction, related to fear that recovery was fragile, with the very real possibility of relapse. Colin was keen to point out change was not a linear process and describes one such relapse below.

*‘There was one occasion when I had half a litre* (cider) *and after that I sat there kicking myself and I’m thinking “Why did you do that?” you know what I mean,”You know, you really don’t need to do it, you don’t need it anymore”.’*

Sam spoke of the fear of difficult periods ahead where he may be tempted to go back to alcohol, commenting on times he may find challenging such as bereavement, and using his own devised plan to cope with such challenges.

*‘Well we’ve had quite a few bereavements recently and that creates pressure, you go to gatherings and there’s drink and it’s a horrible time anyway losing someone and saying goodbye, you know. But I say to myself when things get shaky and you’re in a tight spot there is other ways to turn…and I don’t mean religion or that. I give it 24 h. I don’t give in and that’s really important not to give in.’*

For those who previously identified alcohol as being central to their social lives, sustaining change may be challenging and even lonely: reducing or abstaining from alcohol was clearly not always easy. If times become difficult, there may always be the temptation to return to alcohol and the very real possibility of relapse. As highlighted, many were still embedding behavioural change in their life, continuing to deal with many issues and striking a precarious balance between maintaining positive change and relapsing. These plans were still a work in progress, with employment goals, building a structure for their lives and something to take the place of alcohol all important aims.

## Discussion

The findings from this study assist in understanding the complexity and challenges associated with reducing alcohol consumption. The results consisted of interrelated themes that depicted the participants’ views of alcohol, the ABI and the barriers and enablers to behaviour change. During the qualitative interviews participants spoke freely about the place of alcohol within their lives and their personal perspectives on reducing consumption. There were four super-ordinate themes providing details on a range of issues identified, focusing on 1) personal reflection, 2) ABI/hospital admission, 3) social and psychological cost-benefit analysis, 4) future challenges. Realist evaluation, using the mechanism, context and outcome pattern, acknowledges that interventions such as ABI do not necessarily work for everyone as participants are likely to be embedded in different contexts [17].

Personal reflection, potentially encouraged by the ABI (mechanism), appeared to be important. Participants consciously considered the context of alcohol’s place within their lives with hospital admission providing the space to take stock and consider action. Based on the participants’ narratives, their experiences with alcohol could be described almost in terms of a social and psychological cost-benefit analysis. Participants appeared to weigh up the potential gains against the potential losses in reducing or abstaining from alcohol use (outcome). This brought both perceived positive (social impact, meeting friends at the pub) and negative consequences (impact on relationships and family). Reduced alcohol consumption was not viewed entirely positively by all participants. Some missed the social contact associated with their previous drinking patterns. Previous research supports this finding with alcohol synonymous with relaxation, providing a release from daily pressures, and as a way of obtaining social support and enjoyment [18]. When participants reflected on their future plans, the potential for relapse was identified; it appeared that having a structure, purpose, social support and possibly employment could make the difference between sustaining change and returning to previous drinking patterns. Previous research undertaken around Antonovsky’s Sense of Coherence Theory suggests that having a good reason and purpose within an individual’s life is linked to higher levels of coherence and better health [19, 20]. Occupation and the need to be occupied forms part of human nature, and is a crucial component in overall health and wellbeing [21]. For many of the participants in this study, sustaining the changes they had made appeared to depend on finding something to take the place of alcohol. The importance of a valued occupation links to a previous study on attitudes in Scotland, which identified boredom as a factor linked to alcohol misuse [22].

Some participants in this study explained their previous use of alcohol as a coping mechanism to block or escape from perceived emotional pain. This finding is in keeping with studies that suggest trauma and negative life events may promote coping-orientated alcohol use [23, 24]. Using alcohol to block out emotional pain has been well documented over the past 20 years in clinical and epidemiological samples [25–27].

A significant other who was supportive and who cared enough to take an interest in their alcohol consumption appeared to positively influence levels of intake. Those who gained respect or positive affirmation from others, such as family members, reinforcing that changes were noticeable and worthwhile, appeared more motivated to sustain reduced alcohol consumption. Those experiencing social isolation may look to social situations involving alcohol to fill this gap, potentially leading to excessive consumption [28]. Whilst partners or significant others were not involved in the current study, it is interesting to note that those who reported having a partner supportive of their reduced alcohol consumption appeared better equipped to sustain the adjustments made, whereas those who missed the company and social environment centred on alcohol may find sustaining change more challenging.

The study illustrates the depth and range of experiences, both positive and negative, which influence reduction or abstinence from alcohol and its impact, not only for participants, but also for those around them. Together, the narrative accounts obtained within this study highlight the challenges for a subgroup of participants who reduced or abstained from alcohol after receiving an ABI, and explain the process of change in greater depth than the quantitative findings alone, which are presented separately [3]. The qualitative findings add depth and breadth of knowledge, focusing not only on whether the intervention worked, but adding insight into the process of change and maintenance as well as barriers and facilitators for those who reduced or abstained from alcohol.

The findings from this study appear to concur with the view that hospital admission may represent an opportune time when hazardous/harmful drinkers may be susceptible to exploring changes in their alcohol consumption and supports the idea of hospital admission as a teachable moment [29, 30]. Additionally, the literature suggests that ABIs provide a structure to support and implement this change [9].

### Strengths and limitations

Strengths of the study include the use of semi-structured interviews capturing the lived experience and perspectives of ten hazardous/harmful drinkers who received an ABI and either reduced or abstained from alcohol over a 6 month period. Addressing the experiences of hazardous/harmful drinkers is an aspect that few ABI studies have explored. To enhance the credibility and rigour of the findings, the interviews were transcribed verbatim to allow in-depth analysis of the transcripts. A limitation of this study related to the sample of participants, i.e. inclusion of only those in the intervention arm of the RCT who had reduced their alcohol consumption. This represented only one group of those involved in the trial and it might have been beneficial to interview other subsamples; for example those whose alcohol consumption had not changed, or those in the control group, allowing comparisons to be made between those who reduced or abstained from alcohol and those who did not. Readers should be cautious about the findings as the interviews were undertaken with a small sub-group only within Scotland. Additionally, the sample was a convenience sample and may not have the full range of characteristics representing harmful or hazardous drinkers.

## Conclusions

This study considers some important issues in an era where there is increasing emphasis on how complex interventions work, participants’ experiences and the active ingredients such as what works for whom and in what circumstances [13]. The findings provide an insight into participants’ views on alcohol, ABI, and the barriers and enablers to change. Participants described a social and psychological cost benefit analysis, with some conscious consideration of the advantages and disadvantages of reducing intake or abstaining from alcohol. Findings suggest that, whilst hospital admission can act as a catalyst, encouraging individuals to reflect on their alcohol consumption using ABI may consolidate this, turning this reflective moment into action.

Of particular interest are the findings around enablers and barriers associated with alcohol reduction following an ABI and the context and mechanisms for change. Based on the perspectives from ten harmful/hazardous drinkers, helping people develop change plans appear to be an important component of an ABI and an aspect of particular concern is the potential for relapse. Once individuals decide to change their behaviour, these changes may be difficult to maintain unless they can find activities to take the place of alcohol. For many harmful and hazardous alcohol drinkers, social lives, hobbies, leisure pursuits appear to revolve around alcohol. Potentially, there is a risk that alcohol consumption may rebound to baseline, and further research with additional follow up and/or the provision of booster ABI sessions is worthy of investigation. Having a significant other who took an interest in the individual’s alcohol consumption may influence positive change, and this is acknowledged as an area with potential for further research. Future studies are required to establish the role that occupation plays in maintaining alcohol related behaviour change and breaking the patterns of harmful/hazardous drinking.

## Abbreviations

ABI: Alcohol brief intervention
RCT: Randomised controlled trial
